# The role of proximal body information on anticipatory judgment in tennis using graphical information richness

**DOI:** 10.1371/journal.pone.0180985

**Published:** 2017-07-13

**Authors:** Kazunobu Fukuhara, Hirofumi Ida, Takahiro Ogata, Motonobu Ishii, Takahiro Higuchi

**Affiliations:** 1 Department of Health Promotion Science, Graduate School of Human Health Science, Tokyo Metropolitan University, Minami-Ohsawa, Hachioji, Tokyo, Japan; 2 Department of Sports and Health Management, Jobu University, Toyazuka-Machi, Isesaki, Gunma, Japan; 3 Department of Sport and Medical Science, Teikyo University, Otsuka, Hachioji City, Tokyo, Japan; 4 Department of Human System Science, Tokyo Institute of Technology, Oh-Okayama, Meguro, Tokyo, Japan; Sao Paulo State University, BRAZIL

## Abstract

**Objective:**

Recent studies have reported that skilled tennis players are likely to use proximal body information for anticipating the direction of their opponent’s forehand shot. However, in these studies, the visual stimuli did not include visual information about the ball. Skilled players may have used proximal information owing to the lack of distal information. To address this issue, we developed a novel methodological approach using computer graphics (CG) images in which the entire body was presented by a combination of point-light display (i.e., poor graphical information, PLD) and polygons (i.e., rich graphical information). Using our novel methodological approach, we examined whether skilled tennis players use proximal body information when anticipating shot directions.

**Methods and results:**

Fifteen skilled tennis players and fifteen novice players tried to anticipate shot directions by observing four CG forehand strokes (ALPOL: all body parts were represented with polygon; RAPLD: racket and arm were represented with PLD; BOPLD: body parts without racket and arm were represented with PLD; and ALPLD: all body parts were represented with PLD). Our intention in creating CG models with such combinations (i.e., RAPLD and BOPLD) was that because of the richer graphical information provided by polygons compared to PLD, the participant’s anticipatory judgment would be influenced more by body parts expressed with polygons. The results showed that for skilled players, anticipatory judgment was more accurate when they observed RAPLD than when they observed BOPLD and ALPLD. In contrast, for novice players, there were no differences in the accuracy of anticipatory judgments with the four CG models.

**Conclusions:**

Only skilled players made more accurate anticipatory judgments when body regions were expressed with rich graphical information, and the racket and arm were expressed with poor graphical information. These suggest that skilled players used proximal information to effectively anticipate shot directions.

## Introduction

The ability to anticipate forthcoming events is key to successful athletic performance in racket sports. A number of studies have attempted to understand how tennis players made their anticipatory judgments [1–9]. For example, skilled tennis players outperform novices by anticipating the direction of their opponent’s shots even before their racket made contact with the ball (e.g., [2, 3]). The spatial-occlusion method has been used to obtain critical information on the anticipation skills of tennis [4] and badminton players [10]. In these studies, skilled and novice players observed video clips of shots in which some important reference points, such as the racket, the opponent’s head, or lower body, were occluded. These studies found that, although skilled players generally outperformed their novice counterparts, there were no significant differences in the accuracy of their anticipation when the racket, arm, and ball were obscured. Based on these findings, it was concluded that distal cues associated with the racket-ball contact were the most informative cues to anticipate the direction of forthcoming shots.

In contrast to findings using the spatial-occlusion method, recent studies using a new methodological technique showed that skilled players were also likely to use proximal (i.e., hips, shoulders, and legs) body information when anticipating shot direction [6,7]. In these studies, the kinematic patterns of the opponent’s whole-body movements were presented while the locations of certain parts of the body were distorted according to the results of principal component analysis [5–8]. That is, whereas the studies using the spatial-occlusion method tried to identify the critical cues for anticipation by occluding plausibly important information, these recent studies tried to identify the critical cues for anticipation by distorting them. One of these studies [7] demonstrated that, for skilled players, the accuracy of anticipatory judgments significantly decreased when both distal information (i.e., racket and arm) and proximal information were distorted. In contrast, for novice players, the accuracy of anticipatory judgments significantly decreased only when distal information was distorted. These results suggest that, at the very least, skilled players are likely to use both proximal and distal cues to anticipate the direction of forthcoming shots. This is consistent with a previous study [9] that showed that skilled players fixed their gaze toward the proximal regions of the opponent, probably because the relative motions from the trunk and hip were visible, in order to anticipate the movement of the distal parts (see review for details [11]).

The idea that skilled players were also using proximal body information for anticipation seems reasonable, given that the movement of the proximal body part affects the movement of distal parts (i.e., a kinematic chain between the proximal and distal body parts). At this moment, however, we cannot rule out the possibility that the findings reported in recent studies [6–8] were influenced by methodological characteristics. In these studies, the ball was not displayed. If the spatial relationship between the trajectory of the racket-arm and that of the ball are essential in order to use distal information to anticipate shot direction [4], it is possible that the skilled players in these studies may have used proximal information because of the lack of distal information.

To rule out this possibility, we developed a novel methodological technique in which computer graphics (CG) stimuli of the whole body, the racket, and the ball were displayed. The whole body was presented using a combination of point-light display (PLD) (i.e., presenting minimal kinematic information of body landmark points) (e.g., [12, 13]) and polygon display (i.e., presenting human-like body, such as clothing, skin and limb configuration) (e.g., [14–17]). PLD has sufficient visual information to allow the observer to recognize intentions underlying other people’s actions and such visual perception is widely known as biological motion perception [12, 13]. For example, previous studies have reported that human perception is highly sensitive even to the PLD, in that observers were able to distinguish the gender of a walker (e.g., [18, 19]) and recognize the emotion of an actor (e.g., [20]). Moreover, previous studies on racket sports (e.g., [21, 22]) have reported that skilled players were able to pick up useful advanced cues from PLD. On the other hand, CG models expressed with polygons might elicit superior anticipatory judgments compared to simplified human models, such as PLD and stick figure models [14–17]. The addition of graphical details, such as structural information (e.g., texture, color, contour, and shape) or spatial reference information (e.g., tennis net and line), are likely to enhance the accuracy of anticipatory judgments by skilled and novice tennis players [23].

The combination of CG models between PLD and polygon provide two distinct advantages over two previous methods (i.e., spatial-occlusion and manipulation of kinematic information) to identify the use of advanced cues by skilled tennis players. First, the CG model retains information relevant to the coordination of whole-body movements, as it does not use spatial-occlusion. In particular, polygon display is more suitable in combination with PLD than traditional videos are, because PLD and polygon are based on artificial graphical information generated by computer simulation. Second, the CG model evaluates the accuracy of anticipatory judgment by manipulating the richness of the graphical information regarding the human body shape, instead of manipulating the kinematic information directly. This advantage has a merit that a special technique for manipulating the ball information is not required according to the manipulation of the kinematic information.

Using our novel methodological approach, we examined whether skilled tennis players use proximal body information to effectively anticipate shot directions. We expect that the movements of body parts displayed with polygons are more reliable cues than those displayed with PLD for perceiving the opponent’s movements and anticipating shot directions. Subsequently, if skilled players are using both proximal and distal body information for their anticipation, then it was hypothesized that anticipation by skilled tennis players became inaccurate when the proximal body parts were displayed with PLD.

## Materials and methods

### Participants

Fifteen skilled tennis players (mean (M) ± standard deviation (SD) age of 19.73 ± 0.88 years, 9.73 ± 3.22 years of tennis experience) and 15 novice counterparts (age 18.75 ± 0.68 years) participated in this study. The inclusion criteria ensured that the skilled players were on the university tennis team and have played in national tournaments or top level collegiate competitions. Additionally, all novice participants had played tennis at least once in their life, but did not play regularly. The exclusion criteria ensured that none of participants had visual deficits. The experimental protocol was approved by the institutional ethics committee of Tokyo Metropolitan University and the Declaration of Helsinki. All participants gave their written informed consent prior to participation. None of the participants had previous experience with the experimental task or procedure.

### Visual stimuli (CG images)

**Fig 1:**
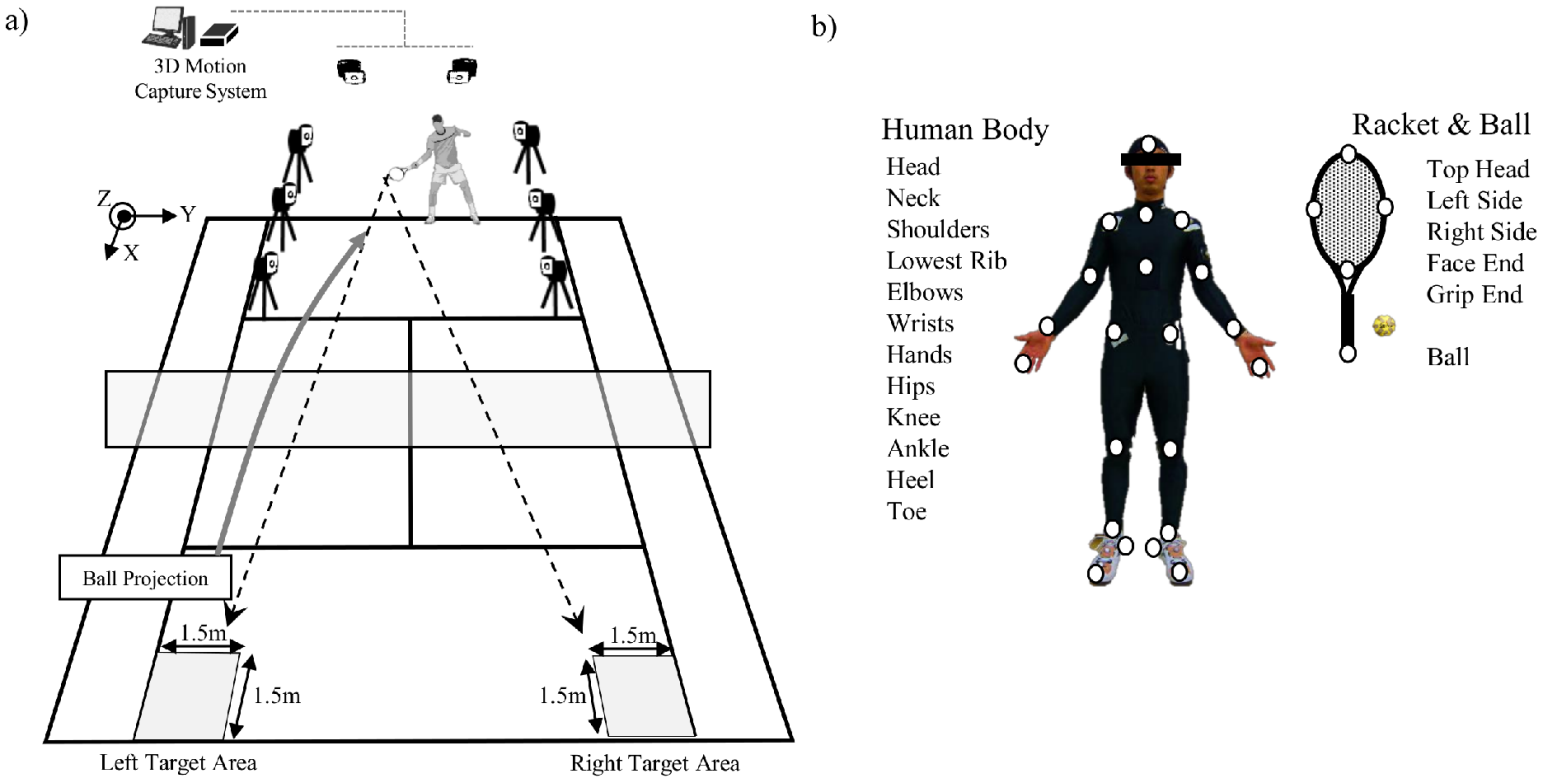
a) Setup for 3-dimensional motion capture of forehand strokes performed by a reference player; the right and left court targets for cross-court and inside-out stroke, respectively, are shown. b) Landmarks used for motion capture, showing 21 anatomical locations on the body and five on the racket and ball.

The protocol for creating CG images, used as visual stimuli for anticipatory judgment, is shown in Fig 1a. First, forehand strokes performed by a professional tennis player were recorded on a tennis court using a three-dimensional motion capture system. The reference player was 22 years old, with 11 years of tennis experience, and was ranked in the top 30 in Japan. Two target areas (1.5 m × 1.5 m) were set on the left side of the court (i.e. inside-out stroke) and on the right side of the court (i.e., cross court stroke). The player was asked to hit the ball using only forehand tennis strokes, with maximum effort, aiming the target areas. The three-dimensional motion analysis system (Hawk system, MotionAnalysis Inc., USA) included eight cameras and tracked forty-one passive retro reflective markers at a sampling frequency of 200 Hz. The positions of 21 anatomical landmarks on the body and five locations on the racket and ball were tracked over each trial (see Fig 1b). To create CG models, we used 14 successful strokes, seven inside-out and seven cross-court strokes.

Secondly, the CG models were constructed with images from motion-capture data, using three-dimensional character animation software (MotionBuilder 2013, Autodesk Inc., USA). To reduce the computational burden of model creation, the motion capture data, obtained at 200 Hz, was resampled at 30 Hz. The basic CG models obtained were subsequently rendered using a three-dimensional CG modeling software (Maya 2013, Autodesk Inc., USA). A black background image was used in the model, which is traditionally used for studies on biological motion perception [13]. The viewpoint of the model was matched to the viewing angle of a receiver positioned at the midpoint of the service line on the tennis court.

**Fig 2:**
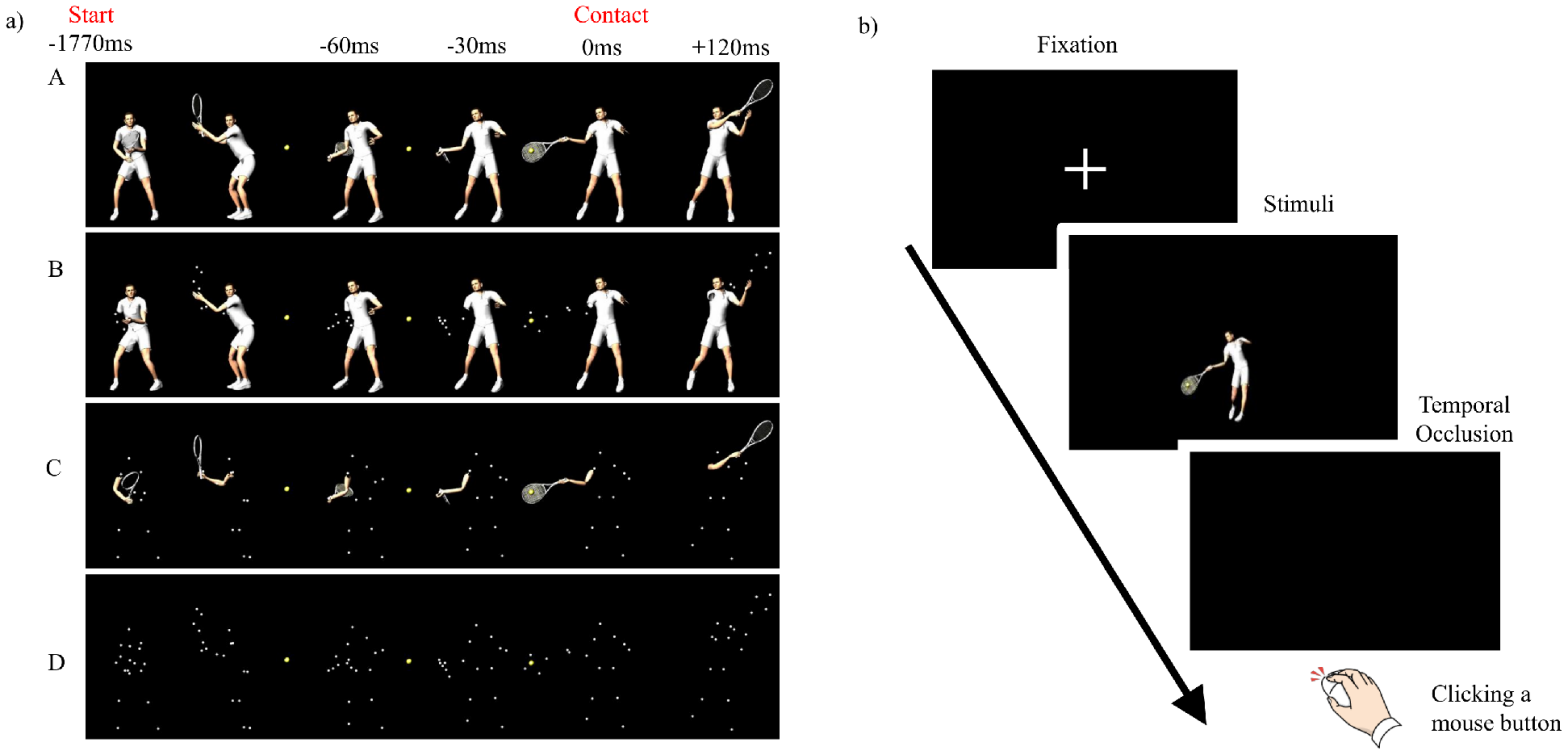
a) The four CG models (A: ALPOL, B: RAPLD, C: BOPLD, and D: ALPLD) with four occlusion timing patterns, relative to ball contact with the racket (-60 ms, -30 ms, 0 ms, and +120 ms). b) Participants were asked to click a mouse button with the right index finger (left side) or a middle finger (right side) to indicate the anticipated direction of the ball.

Thirdly, the following four types of CG images were created from each of the basic models (Fig 2a, S1 Movie):

1. ALPOL (all polygon) model: graphical information was presented with polygons.
2. RAPLD (racket-arm PLD) model: graphical information on racket-arm regions were presented with PLD, whereas the information on other regions was presented with polygons.
3. BOPLD (body PLD) model: graphical information on all the body regions except for the racket and arm regions were presented with PLD, whereas the information on the racket and arm regions were presented with polygons.
4. ALPLD (all PLD) model: graphical information was presented with PLD.

Finally, the length of exposure to each CG model was manipulated to achieve four different temporal occlusion patterns (e.g., 3, 22): occlusion of visual information for -60 ms and -30 ms prior to ball contact, at the moment of ball contact (i.e., 0 ms), and for 120 ms after ball contact. We selected the 120ms occlusion condition to confirm whether skilled and novice players used ball-flight information after the contact for anticipatory judgments. In total, we created 244 video clips for analysis: 14 shots × 4 CG models × 4 temporal occlusion patterns.

### Anticipatory judgment task

Participants sat on a chair with their heads fixed on a chin support. The visual stimuli were projected (W1080ST, BenQ, Taiwan; 1920 × 1080 resolution) onto a large (1.77m × 0.99 m) screen positioned 3 m in front of participants. The CG tennis player was adjusted to 0.3 m in height from the foot to the top of the head. A presentation software (E-prime 2.0, Psychological Software Tools Inc., USA) was used to present the visual stimuli and collect participants’ responses. The vertical visual angle was approximately 5.42 degrees, which was similar to that of a receiver on the tennis court. Participants were instructed to watch the visual stimulus presented and to anticipate the direction of the ball to the left or right target areas. We did not set a time constraint for responding, but asked the participants to response as soon as the stimulus was occluded by clicking the corresponding mouse buttons for the left and right targets (Fig 2b). Prior to testing, participants completed 10 practice trials (5 left and 5 right shots trials, randomly presented) to familiarize themselves with the task procedure. For the testing session, participants completed 244 trials (i.e., response to all visual stimuli was measured once). Trials were divided into four blocks of 56 trials each (14 strokes × 4 CG models). The timing of temporal occlusion was the same within each block, with the order of blocks was counterbalanced. The 14 shots and 4 temporal occlusions in a block were randomized. Additionally, no trial in practice or test provided feedback about correct and wrong judgments. A 5-min rest period was provided between blocks and the total session was approximately 60 min in duration.

### Data analysis

The dependent variable was the percentage of correct responses for shot direction. An arcsine transformation was applied to the dataset of correct scores to satisfy the normal distribution assumption. The transformed scores were analyzed using a three-way factorial analysis of variance (ANOVA) with the groups (skilled and novice) used as the between-participants factors, and the CG models (ALPOL, RAPLD, BOPLD, and ALPLD) and temporal occlusions (-60 ms, -30 ms, 0 ms, and +120 ms) as the within-participants factors. Then, in order to focus on the effects of rich graphical information (CG models × temporal occlusion) within each skill group, a planned two-way ANOVA for the separate skill group was also performed.

The significant main and interaction effects were evaluated using Bonferroni-corrected pairwise comparisons. Partial eta-squared (η_p_^2^) values provided a measure of effect size. The significance level was set at α = 0.05. To investigate whether the percentage of correct responses exceeded 50% guessing level (chance level), one-sample *t*-tests were also performed to evaluate the percentage of correct responses under each experimental condition.

## Results

### One-sample t-tests

**Fig 3:**
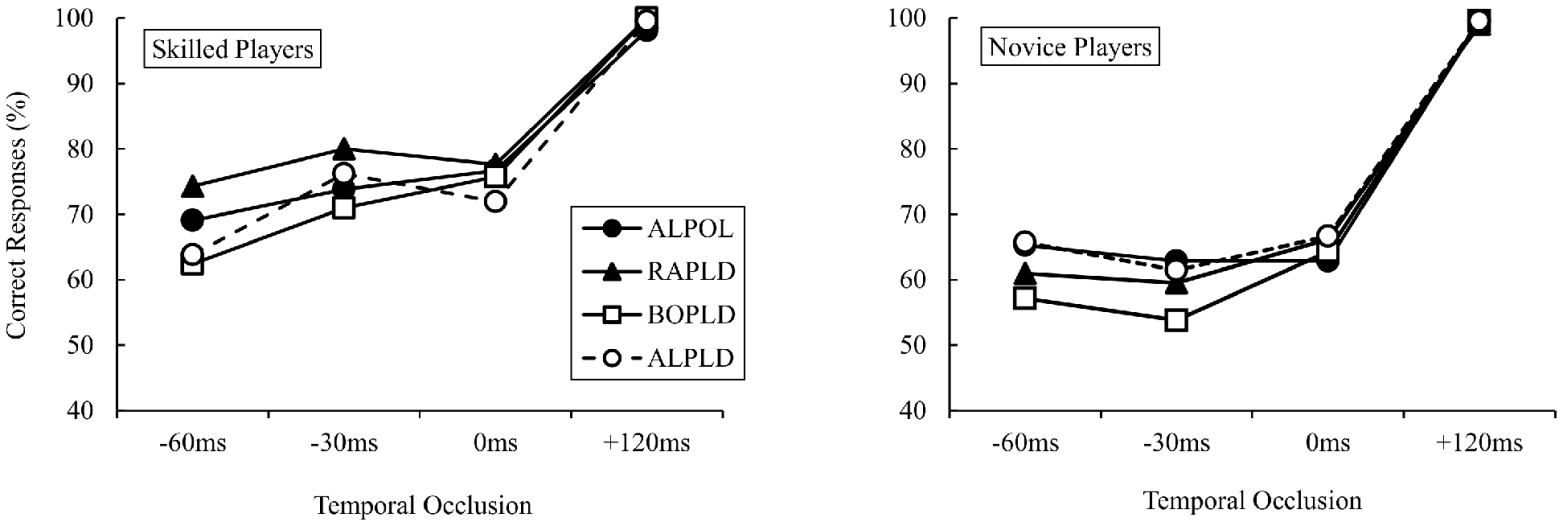
The mean of accuracy scores (skilled and novice players) for the four CG model for each of the four temporal occlusion conditions, with the chance level set at 50%.

The mean percentage of correct responses for skilled and novice participants are shown in Fig 3 (see details in S1 Data). For the skilled participants, the percentage of correct responses was significantly greater under all conditions, with the rate of correct responses above the 50% level of chance for all conditions (*p* < 0.01). For the novice participants, the percentage of correct responses exceeded the level of chance for all conditions (*p* < 0.01), with the exception of the -30 ms occlusion condition for the BOPLD model.

### Model manipulation tests

First, an overall three-way ANOVA (group × CG models × temporal occlusions) on the percentage of correct responses was employed to test the effect of the factors (see the Data Analysis). Then, since a part of the accuracy of the novice group was not above the chance level, a planned two-way ANOVA (CG models × temporal occlusions) was also conducted in order to focus on the effect of rich graphical information within each skill group.

The mean percentage of correct responses under each experimental condition for the skilled and novice groups are shown in Fig 3. A three-way ANOVA identified a significant main effect for CG models (*F* (3, 84) = 2.87, *p* < 0.05, η_p_^2^ = 0. 09). Post-hoc analyses indicated that there were no significant differences among the CG models (*p* = 0.10). The main effect for each group was significant (*F* (1, 28) = 2.81, *p* < 0.01, η_p_^2^ = 0.50), with a higher percentage of correct responses for the skilled (M = 79.38%, SD = 16.15) than for the novice (M = 71.52%, SD = 19.26) groups. The main effect of occlusion patterns was significant (*F* (3, 84) = 507, *p* < 0.01, η_p_^2^ = 0.95). Post-hoc analyses indicated a greater percentage of correct responses for the +120 ms occlusion condition than for other temporal occlusion conditions (all *p* < 0.01) (S1 Fig). In addition, accuracy was greater for the 0 ms occlusion pattern than for the -60 ms occlusion (*p* < 0.05).

A significant group × temporal occlusions interaction was identified (*F* (3, 84) = 9.37, *p* < 0.01, η_p_^2^ = 0.25), indicating that group differences were dependent on the timing of the occlusions (S2 Fig). The percentage of correct responses was greater for the skilled group for all but the +120 ms occlusion condition (-60 ms, *p* < 0.05, -30 ms and 0 ms, *p* < 0.01). Moreover, in the skilled group, the percentage of correct responses was also significantly different between each pair of temporal occlusion conditions (-60ms vs 0ms, *p* < 0.05, and other each pair, *p* < 0.01), except between -30 ms and 0 ms. For the novice group, the percentage of correct responses was greater under the +120 ms occlusion condition than for all other conditions (all *p* < 0.01).

A significant group × CG models interaction (*F* (3, 84) = 2.87, *p* < 0.05, η_p_^2^ = 0.09) indicated that the skilled participants were more accurate than their novice counterparts for all model conditions (ALPOL, RAPLD, BOPLD: *p* < 0.01), but no significant difference between-group was found for the ALPLD model (*p* = 0.07) (S3 Fig). However, the CG models × temporal occlusion interaction (*F* (9,252) = 1.44, *p* = 0.17, η_p_^2^ = 0.05) and CG models × temporal occlusion × group interaction (*F* (9,252) = 0.97, *p* = 0.47, η_p_^2^ = 0.03) were not significant.

A planned two-way ANOVA for the skilled group identified a significant main effect for the CG models (*F* (3, 42) = 3.92, *p* < 0.05, η_p_^2^ = 0.22). Post-hoc analyses indicated a greater percentage of correct responses for the RAPLD model than for the BOPLD and ALPLD models (all *p* < 0.05) (S3 Fig). There were no significant interactions for the CG models × temporal occlusions (*F* (9, 126) = 1.44, *p* = 0.17, η_p_^2^ = 0.09). On the other hand, a planned two-way ANOVA for the novice group showed no significant main effect for the CG models (*F* (3, 42) = 1.89, *p* = 0.15, η_p_^2^ = 0.12). There was no significant interaction for the CG models × temporal occlusions (*F* (9, 126) = 0.91, *p* = 0.51, η_p_^2^ = 0.06).

## Discussion

This study examined whether skilled tennis players use proximal body information to effectively anticipate shot directions using manipulation of graphical information richness. We employed a novel methodological approach in which the opponent’s forehand strokes were presented as CG models created with a combination of PLD and polygons. The intention in creating CG models with this combination was that, because of the richer graphical information provided by polygons compared to PLD, participants’ anticipatory judgment would be influenced more by the body parts expressed with polygons.

Before discussing the main findings on the effect of the CG models, it is important to emphasize that several findings in the present study showed the validity of using CG models for investigating anticipatory judgment in racket sports. First, the percentage of correct responses was significantly above chance levels (50%) for almost all conditions (except for the novice player’s ALPLD model condition with a −30ms temporal occlusion). This is comparable to previous studies that used videos (e.g., [1, 4]), PLD or stick figure images (e.g., [6–9]), and CG images [15–17]. This suggests that our unique CG models were successful in providing participants with useful advanced cues to anticipate shot directions. Second, the present study successfully replicated the previous findings of related studies in terms of (a) the advantage of skilled players over novice players with regard to general anticipatory performance (e.g., [1–4]) and (b) the advantage of skilled players when the opponents' movement was temporally occluded prior to racket–ball contact in racket sports (e.g., [3, 21, 22]). Moreover, the percentage of correct responses was a ceiling effect (about 100%), and no advantage on the skilled players could be seen when the ball flight information after the contact was included. From these findings, we can safely say that our unique CG models compare favorably with the use of videos or other types of CG images to investigate anticipatory judgment in tennis.

The main finding was that, for skilled players, anticipatory judgment was more accurate with the RAPLD model than with the BOPLD and ALPLD models. In the RAPLD model, proximal body parts were expressed with polygons, while the distal parts were expressed with PLD. In contrast, in the BOPLD and ALPLD models, proximal body parts were expressed with PLD, while the distal parts were provided with polygons. The characteristics of these models suggest that skilled players showed more accurate anticipation when the graphical information on proximal body parts was richer.

These findings are partially consistent with previous studies [6, 7] in that the information on proximal body parts was used for anticipation by skilled players. However, our findings did not support the idea that both proximal and distal body parts were used for anticipation. The discrepancy between our present study and previous studies may be attributed to the fact that, in our study, accurate kinematic information was provided even when a body part was expressed with polygons. This clearly differs from previous studies where no kinematic information was provided with the spatial-occlusion method [4, 10, 19], and where kinematic information was distorted [5–8]. Due to this factor, the attention of skilled players may have been focused on the body parts expressed with polygons while the other body parts were expressed with PLD.

We did not find any advantage for the ALPOL model, in which the entire body was represented by polygons. However, ALPLD model was also not significant (*p* = 0.07) between the skill groups. These results suggest that the kinematic information provided to the participants did not clearly differentiate between the polygon and PLD, and our data did not support the idea that skilled players were able to pick up useful advanced cues from PLD compared to novice players (e.g., [10, 21, 22]). Previous studies [21, 22] reported that the correct responses in PLD are significantly lower than that in the videos. One of these factors is considered to be due to the lack of additional graphical details, such as structural information (e.g., texture, color, contour, and shape) or spatial reference information (e.g. tennis net and line) [23]. In general, human observers have a lot of perceptual experience regarding visual information in actual human shape [14]. Such perceptual familiarity may have the effect of directing the attention of skilled players to graphical information with polygons.

If this explanation holds true, then it follows that skilled players rely on proximal body parts for anticipatory judgment. Given that the movement of the proximal body part affects the movement of distal parts (i.e., a kinematic chain between the proximal and distal body parts), the use of proximal body information was effective to anticipate the movement of distal body parts, which critically affects shot direction. In other words, skilled players can anticipate forthcoming shot directions much earlier when using the kinematic information of proximal body parts than when using the kinematic information of distal body parts.

We believe that the characteristics of our methodology can also explain the finding that the accuracy of anticipatory judgment by novice players was not significantly different among the CG model conditions. Because the kinematic information of distal body parts was available even when these body parts were expressed with polygons, novice players may have always used the kinematic information of distal body parts for their anticipation.

Previous studies [6, 7] suggested that skilled tennis players adopted the global perceptual strategy (i.e., the use of distal and proximal cues), while novice players depended exclusively on the local strategy (i.e., the use of only distal cues). Although our findings showed the importance of proximal body movement for anticipation, we believe that these are partially consistent with previous studies because the use of proximal body information was available to anticipate distal body movement. Future studies are necessary to test the validity of these explanations.

### Limitation

There are some limitations in this research method. The reference model in this study was a professional tennis player with sophisticated actions who could accurately target two areas. The number of shots was 14. However, this information is relatively smaller than other previous studies on racket sports (e.g. [5–9]). It is necessary to examine whether similar results can be obtained even if other reference models and different movement patterns are used. Another limitation is that we did not clarify the influence of the temporal effect among the CG models using a temporal occlusion approach. The polygon may be processed faster than PLD because human observers have considerable perceptual experience regarding actual human shape [14]. On the other hand, PLD may be processed faster than the polygon because it has no additional graphical details and it preserves kinematic patterns for visual recognition [13]. Future studies will be needed to solve the problem of temporal information processing using reaction time task.

## Conclusion

We introduced a novel methodological approach in which CG models created with a combination of PLD and polygons were used to identify whether skilled tennis players used proximal body information to anticipate their opponent’s shot directions. Result showed that skilled players used proximal information when the body regions were expressed with rich graphical information (i.e., polygons). In contrast, novice players' anticipatory judgments were not affected by the richness of graphical information on bodily cues. These results suggest that skilled players use proximal information to effectively anticipate their opponent’s shots. Our novel methodological approach may be proposed as a new evaluation method to identify spatiotemporal information sources underlying skilled player’s anticipatory judgment in tennis.

## Supporting information

S1 Movie An example of four types of CG images. (WMV)

S1 Data All data used in statistical analysis in Fig 3. (CSV)

S1 Fig The mean and standard deviation of accuracy scores for each of four temporal occlusion conditions. (TIF)

S2 Fig The mean and standard deviation of accuracy scores (skilled and novice players) for each of four temporal occlusion conditions. (TIF)

S3 Fig The mean and standard deviation of accuracy scores (skilled and novice players) for each of four CG models. (TIF)
