# Supplementary figures and images for: The role of proximal body information on anticipatory judgment in tennis using graphical information richness
Source: PLoS One. 2017 Jul 13;12(7):e0180985. doi: 10.1371/journal.pone.0180985 (PMC5509252; doi:10.1371/journal.pone.0180985)

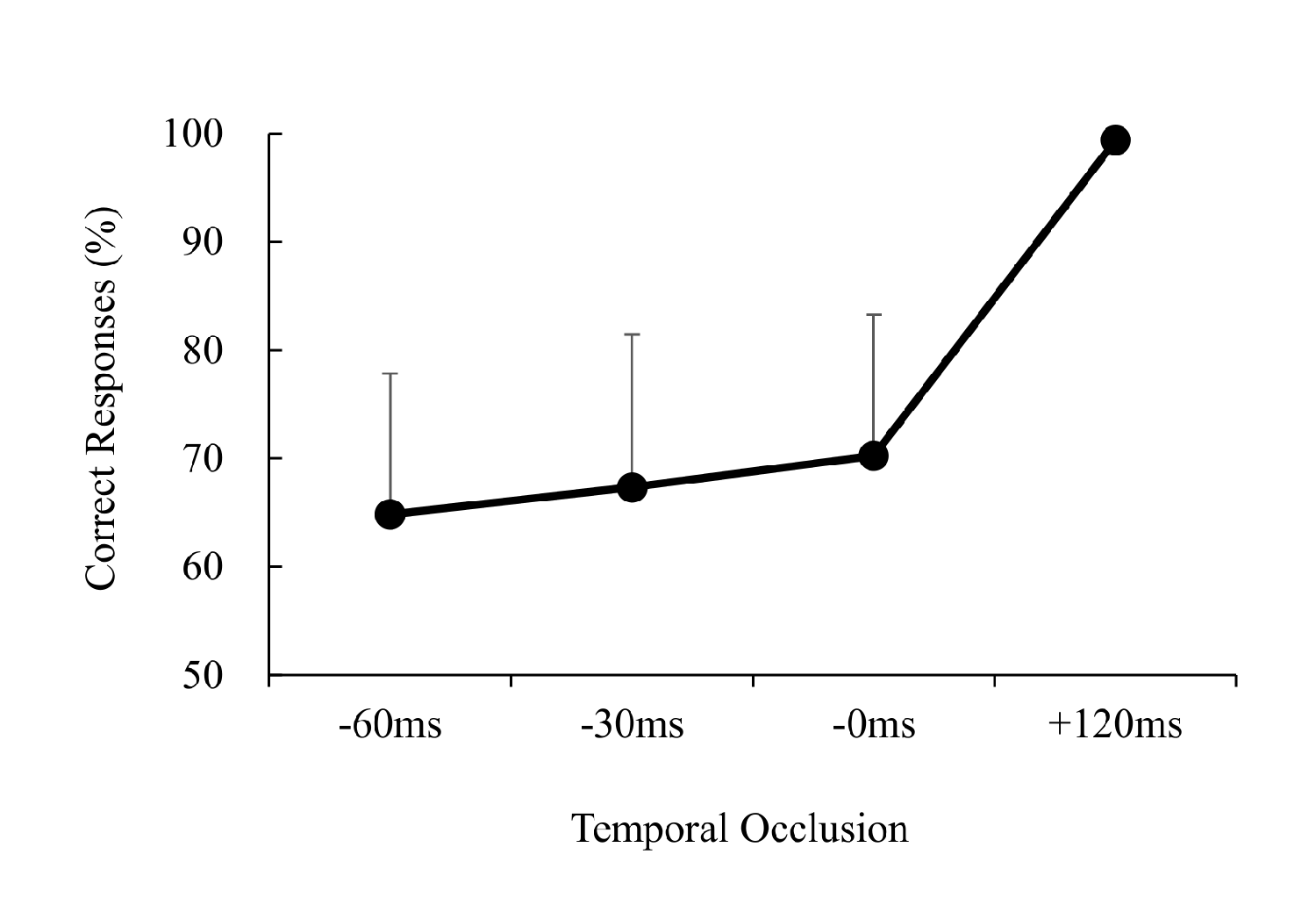

Supplement: S1 Fig — (TIF) [file pone.0180985.s003.tif]

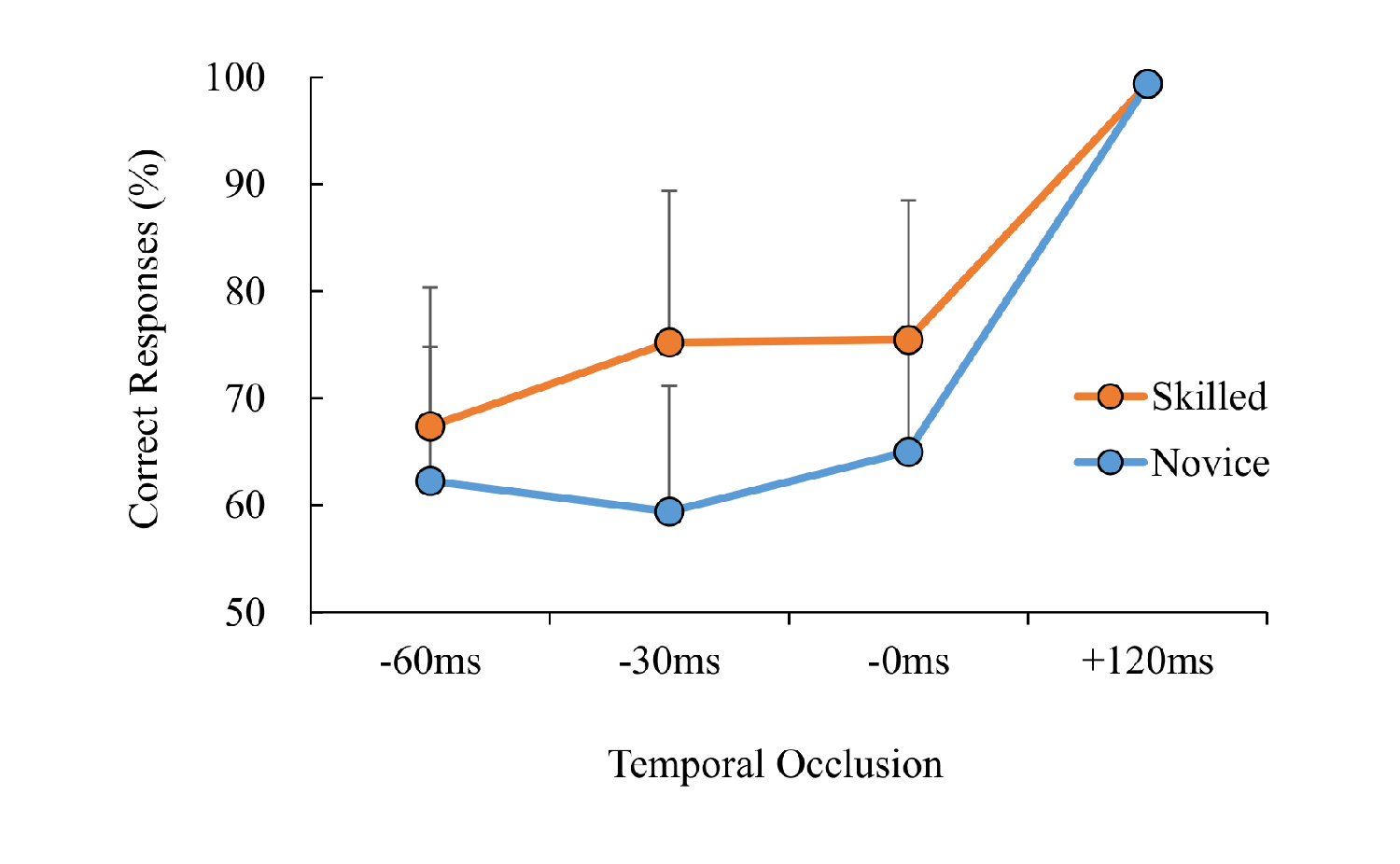

Supplement: S2 Fig — (TIF) [file pone.0180985.s004.tif]

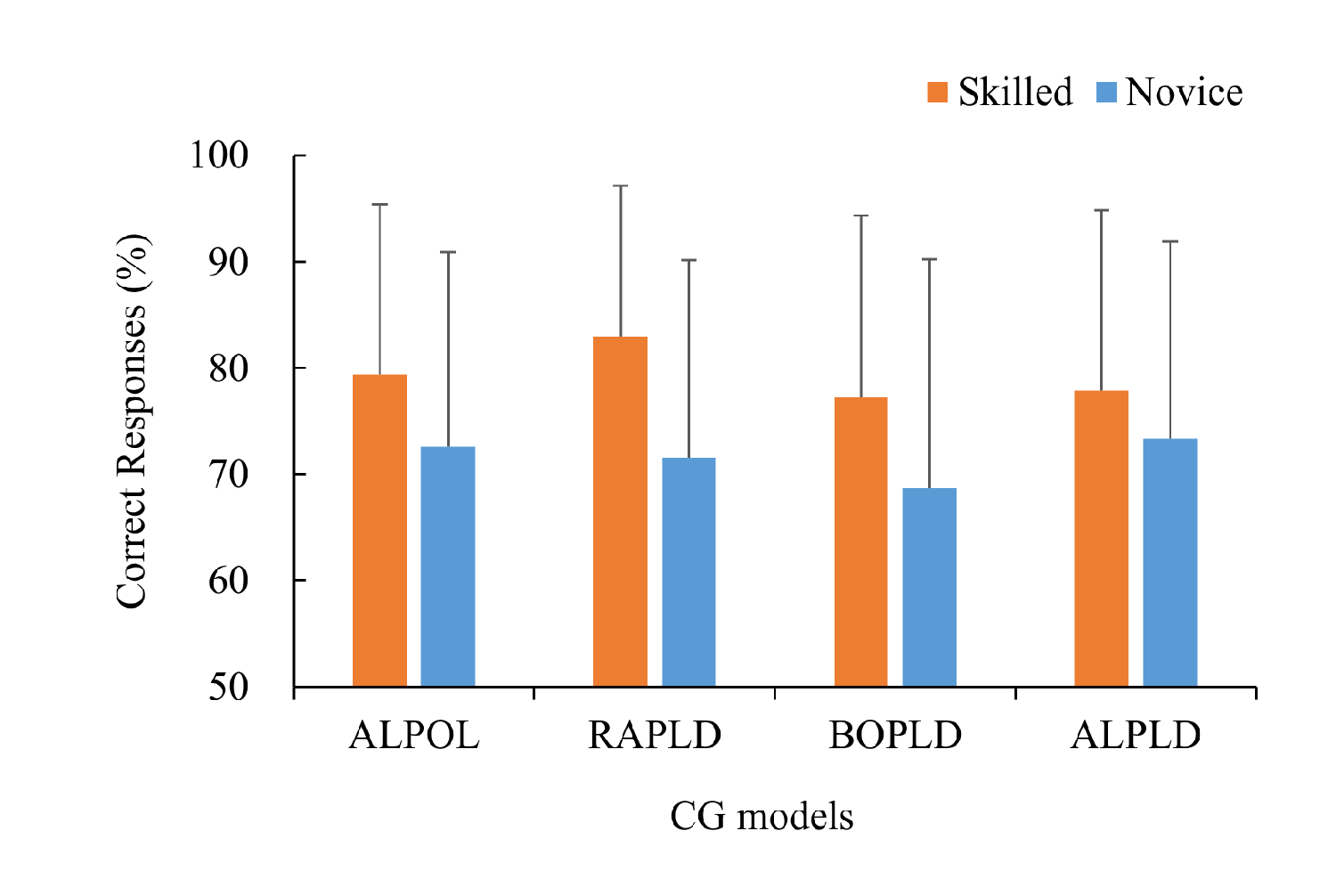

Supplement: S3 Fig — (TIF) [file pone.0180985.s005.tif]
